# Low Plasma Levels of Soluble Endoglin and Cardiovascular Events in Patients Undergoing Coronary Angiography

**DOI:** 10.3390/biomedicines11112975

**Published:** 2023-11-04

**Authors:** Emi Saita, Yoshimi Kishimoto, Masayuki Aoyama, Reiko Ohmori, Kazuo Kondo, Yukihiko Momiyama

**Affiliations:** 1 Research Institute of Environmental Medicine, Nagoya University, Furo-cho, Chikusa-ku, Nagoya 464-8601, Japan; 2 Department of Food Science and Human Nutrition, Faculty of Agriculture, Setsunan University, 45-1 Na-gaotouge-cho, Hirakata 573-0101, Japan; 3 Department of Cardiovascular Medicine, Toho University Graduate School of Medicine, 5-21-16 Omorinishi, Ota-ku, Tokyo 143-8540, Japan; 4 Department of Cardiology, National Hospital Organization Tokyo Medical Center, 2-5-1 Higashigaoka, Meguro-ku, Tokyo 152-8902, Japan; 5 Faculty of Regional Design, Utsunomiya University, 350 Minecho, Tochigi 321-8505, Japan; 6 Ochanomizu University, 2-1-1 Otsuka, Bunkyo-ku, Tokyo 112-8610, Japan

**Keywords:** atherosclerosis, biomarker, cardiovascular events, coronary artery disease, endoglin

## Abstract

TGF-β is recognized as playing a protective role against atherosclerosis. Endoglin is a receptor for TGF-β, and its expression is upregulated in atherosclerotic plaques. Endoglin is secreted from the cell membrane into the circulation as a soluble form (sEng). We previously reported that plasma sEng levels were low in patients with coronary artery disease (CAD). However, the prognostic value of sEng levels has not been clarified. We investigated the association between plasma sEng levels and cardiovascular events in 403 patients who had an elective coronary angiography and were then followed up. Cardiovascular events were defined as cardiovascular death, myocardial infarction, unstable angina, heart failure, stroke, or coronary revascularization. Of the 403 patients, 209 (52%) had CAD. Plasma sEng levels were lower in patients with CAD than in those without CAD (median 4.26 vs. 4.41 ng/mL, *p* < 0.025). During a mean follow-up period of 7.5 ± 4.5 years, cardiovascular events occurred in 79 patients. Compared with 324 patients without events, 79 with events had lower sEng levels (3.95 vs. 4.39 ng/mL) and more often had an sEng level < 3.9 ng/mL (47% vs. 28%) (*p* < 0.02). A Kaplan–Meier analysis showed lower event-free survival in patients with sEng < 3.9 ng/mL than in those with ≥3.9 ng/mL (*p* < 0.02). In a multivariate Cox proportional hazards analysis, the sEng level (<3.9 ng/mL) was an independent predictor of cardiovascular events (hazard ratio: 1.59; 95%CI: 1.01–2.49). Furthermore, only among the 209 patients with CAD, the sEng level was also a predictor of further cardiovascular events (hazard ratio: 2.07; 95%CI: 1.24–3.45). Thus, low plasma sEng levels were found to be associated with an increased risk of cardiovascular events in patients with CAD and patients undergoing coronary angiography.

## 1. Introduction

Transforming growth factor-β (TGF-β) is a multi-functional cytokine, and the TGF-β signaling pathway contributes to a wide range of immunological and biological effects on various cell types; however, the precise function of TGF-β in atherosclerosis remains controversial [1]. However, TGF-β is considered at present to play a protective role against the progression of atherosclerosis due to its anti-inflammatory effect as well as its inhibitory effect on the migration and proliferation of vascular endothelial cells, smooth muscle cells (SMCs), and macrophages [2]. TGF-β has been shown to inhibit E-selection expression and inflammatory responses in human endothelial cells [3]. In apoE-deficient mice, TGF-β inhibition was demonstrated to accelerate inflammation and atherosclerosis associated with plaque instability and reduced collagen content [4]. 

Endoglin, also called TGF-β receptor III or CD105, is a homodimeric transmembrane glycoprotein receptor for TGF-β that regulates TGF-β signaling [5]. Endoglin can bind TGF-β1 and TGF-β3 and form a functional receptor complex [6]. The inhibition of endoglin has been shown to enhance TGF-β1 signaling to suppress the migration and proliferation of cultured human vascular endothelial cells [7]. The interference with endoglin has been shown to promote the migration and proliferation of endothelial cells and to inhibit their apoptosis [8]. However, decreased endoglin expression has also been reported to be associated with lower NO-dependent vasodilation and reduced eNOS expression [9]. Endoglin expression is upregulated during wound healing and in inflammatory tissues, and its expression in blood vessels is increased during hypoxia or following vascular injury [1]. Endoglin expression has been shown to be undetectable in normal vessel walls; however, it is upregulated in vascular SMCs of human atherosclerotic plaques [10]. Furthermore, the high expression of both endoglin and TGF-β has also been reported in endothelial cells, SMCs, and macrophages of human atherosclerotic aortic tissues [11]. Notably, high endoglin expression was shown to be associated with increased collagen, increased SMCs, and decreased intraplaque thrombi in human carotid atherosclerotic plaques, thus suggesting a role of endoglin in plaque stabilization [12]. However, the effect of endoglin on the progression of atherosclerosis is still controversial.

Endoglin is released from the cell membrane into the blood as a soluble form (sEng), which is generated by the cleavage with membrane-type metalloprotease-14 (MMP-14) that is abundantly expressed in endothelial cells [5]. The role of sEng is considered to be a naturally occurring antagonist of TGF-β, and increased sEng levels may reduce TGF-β signaling, leading to enhanced atherosclerosis [5]. Notably, we previously investigated plasma sEng levels in 244 patients undergoing elective coronary angiography for suspected coronary artery disease (CAD) and reported that sEng levels were lower in patients with CAD than in those without CAD and were inversely associated with the presence and severity of CAD [13]. However, blood sEng levels in patients with CAD remain controversial [14,15], and their prognostic value has not been fully clarified yet. Therefore, to elucidate the association between plasma sEng levels and cardiovascular events, the present study expands upon our previous report [13] by increasing the number of study patients (from 244 to 403 patients) and following up for cardiovascular events.

## 2. Materials and Methods

### 2.1. Study Patients

From July 2008, we prospectively collected blood samples and clinical and angiographic data from patients undergoing coronary angiography at Tokyo Medical Center. Patients with a history of percutaneous coronary intervention (PCI) or coronary artery bypass grafting (CABG) or those on hemodialysis were not asked to participate in our study. A total of 658 consecutive patients underwent an elective coronary angiography for suspected CAD because of symptoms, such as chest pain and dyspnea on exertion, and electrocardiographic abnormalities from July 2008 to November 2013. Of the 658 patients, 172 patients with acute coronary syndrome (ACS), such as acute myocardial infarction (MI) and class III unstable angina, defined by the Braunwald’s classification [16], were excluded from the present study. Since serum sEng levels were reported to be high in patients with suspected left ventricular (LV) dysfunction [17], 52 patients with any history of heart failure (HF) were also excluded. Since endoglin was shown to be abundantly expressed in prostate, breast, and colon cancers [18], 8 patients with any cancer were excluded. Moreover, 23 patients whose blood samples had run out were excluded. As a result, we investigated the association between plasma sEng levels and cardiovascular events in 403 patients undergoing elective coronary angiography. This study was approved by the institutional research ethics committee of our hospital (Approval No. R08-050/R21-037). After obtaining written informed consent following the Declaration of Helsinki, overnight-fasting blood samples were collected on the morning of the day of coronary angiography. No blood sample was collected after the administration of heparin. We defined hypertension as blood pressure levels ≥140/90 mmHg or on medication; 243 patients (60%) were on anti-hypertensive drugs. We also defined hyperlipemia as LDL cholesterol >140 mg/dL or on medication; 144 patients (36%) were on statin. Diabetes mellitus (DM) was defined as fasting glucose levels ≥126 mg/dL or on treatment; 99 patients (25%) were diabetic. Smoking was defined as ≥10 pack years; 173 (43%) were smokers.

### 2.2. Measurements of Plasma sEng and C-Reactive Protein (CRP) Levels

Blood samples were extracted into EDTA-containing tubes and then centrifuged at 2000× *g* for 15 min at 4 °C. The plasma was frozen and then stored at −80 °C until the analysis. As previously reported [13], plasma sEng levels were measured using an enzyme-linked immunosorbent assay (ELISA) with a commercially available kit (Human Endoglin, R&D Systems, Minneapolis, MN, USA) according to the manufacturer’s instructions. According to the data supplied by the manufacturer, the lowest sensitivity of this assay was 0.03 ng/mL, and its measuring range was from 0.16 to 10 ng/mL. The intra-assay and inter-assay coefficients of variation were <3.2% and <6.7%, respectively. Plasma high-sensitivity CRP (hsCRP) levels were also measured by a BNII nephelometer (Dade Behring, Tokyo, Japan). 

### 2.3. Baseline Coronary Angiography and Clinical Follow-Up Results for Cardiovascular Events

Coronary angiography was performed with the Philips Electronics cine angiogram system. On angiograms, CAD was defined as at least one coronary artery with >50% luminal diameter stenosis, and the severity of CAD was represented as the number of vessels with >50% stenosis. The degree of coronary stenosis in each segment by the Coronary Artery Surgery Study classification was evaluated by a visual assessment and then divided into 5 grades (<25%, 26–50%, 51–75%, 76–90%, and >90% stenosis). All angiograms were evaluated by a single cardiologist (Y.M.), who had been blinded to the clinical and laboratory data. The LV systolic function was assessed as an LV ejection fraction (LVEF) determined by echocardiography. All the study patients were followed up for 7.3 ± 4.5 years for cardiovascular events, which were defined as cardiovascular death, myocardial infarction (MI), hospitalization for unstable angina, HF or stroke, or the need for coronary revascularization (PCI or CABG). However, if the PCI or CABG was planned and then performed as a result of the baseline angiography, they were not considered as cardiovascular events. The clinical outcomes were evaluated by reviewing the patients’ medical records.

### 2.4. Statistical Analysis

For parametric, nonparametric, and categorical variables, an unpaired *t*-test, the Mann–Whitney U test, and the chi-squared test were used to evaluate the differences between two groups, respectively. Parametric and categorial variables are shown as mean ± SD and number and percentage (%), respectively. Since the measured sEng and hsCRP levels were considered to be nonparametric variables based on the Shapiro–Wilk test, these results were presented as the median value and interquartile range. The optimal cutoff point of sEng level for cardiovascular events was determined to be 3.9 ng/mL, which showed the highest Youden index. The event-free survival rates for patients with sEng level of <3.9 ng/mL and those with ≥3.9 ng/mL were compared by the Kaplan–Meier method with a log-rank test. Regarding the cutoff point of hsCRP levels, the previously reported cutoff point of 1.0 mg/L for cardiovascular events was used [19,20]. A multivariate Cox proportional hazards regression analysis was used to identify the independent predictors of cardiovascular events. To evaluate the added predictive value of sEng levels for cardiovascular events, a C-statistics analysis was performed. Receiver operating characteristic (ROC) curves were created from multivariate regression models, and the areas under the ROC curves (C-statistics) were measured and compared. We conducted all the statistical analyses using the IBM SPSS software package (ver. 25, Tokyo, Japan), and a *p* value of <0.05 was considered to indicate statistical significance. 

## 3. Results

Of the 403 study patients, 209 (52%) patients were found to have CAD, of whom 110 and 38 patients underwent PCI and CABG, respectively, as a result of the baseline coronary angiography. However, 61 patients with CAD selected only medical treatment over either PCI or CABG after the discussions with the physicians in charge of them. Compared with 194 patients without CAD, 209 patients with CAD had higher plasma hsCRP levels (median 0.80 vs. 0.53 mg/L, *p* < 0.01). In contrast, plasma sEng levels were significantly lower in patients with CAD than in those without CAD (median 4.26 vs. 4.41 ng/mL, *p* < 0.025) (Table 1).

During a mean follow-up period of 7.3 ± 4.5 years, cardiovascular events occurred in 79 (20%) patients (cardiovascular death, n = 11; MI, n = 5; unstable angina, n = 8; HF, n = 22; stroke, n = 10; coronary revascularization, n = 23). Compared with 324 patients who had no events, 79 patients who developed cardiovascular events were significantly older (70 ± 11 vs. 66 ± 11 years) and had lower HDL cholesterol (51 ± 14 vs. 55 ± 15 mg/dL) and higher hsCRP (median 0.82 vs. 0.60 mg/L) levels (*p* < 0.05) levels. Furthermore, patients with events had a higher rate of CAD (78% vs. 45%), a greater number of stenotic vessels (1.7 ± 1.1 vs. 0.8 ± 1.0), and lower LVEF (61 ± 12 vs. 63 ± 9%) (*p* < 0.05) (Table 2).

**Figure 1:**
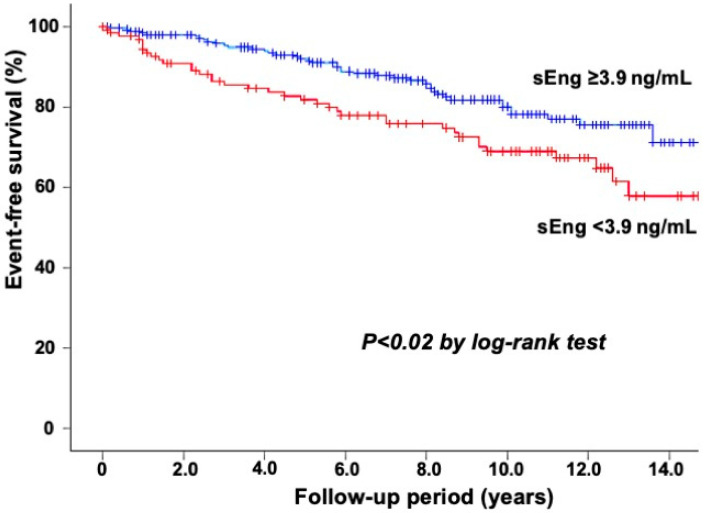
The event-free survival from cardiovascular events in the 403 study patients. The Kaplan–Meier analysis demonstrated a lower event-free survival rate in patients with sEng level <3.9 ng/mL than in those with ≥3.9 ng/mL (*p* < 0.02).

Notably, patients with cardiovascular events had significantly lower sEng levels than those without events (median 3.95 vs. 4.39 ng/mL) and more often had an sEng level of <3.9 ng/mL (47% vs. 28%) (*p* < 0.02). To clarify the association between sEng levels and cardiovascular events, the 403 study patients were divided into two groups based on their sEng levels (<3.9 and ≥3.9 ng/mL). A Kaplan–Meier analysis showed a lower event-free survival rate in patients with an sEng level of <3.9 ng/mL than in those with ≥3.9 ng/mL (*p* < 0.02) (Figure 1). In the multivariate Cox proportional hazards analysis, sEng level as well as CAD and LVEF were found to be independent predictors of cardiovascular events, but the hsCRP level was not. The hazard ratio (HR) of the sEng level <3.9 ng/mL for cardiovascular events was 1.59 (95%CI: 1.01–2.49, *p* < 0.05) (Table 3). In the C-statistics analysis, the addition of sEng levels to the model including age, sex, hypertension, hyperlipidemia, stain use, smoking, diabetes mellitus, CAD, coronary revascularization at baseline, LVEF, and HDL cholesterol and hsCRP levels increased the C-statistics from 0.71 (95%CI: 0.65–0.77) to 0.73 (95%CI: 0.67–0.79). However, this improvement did not achieve statistical significance.

**Figure 2:**
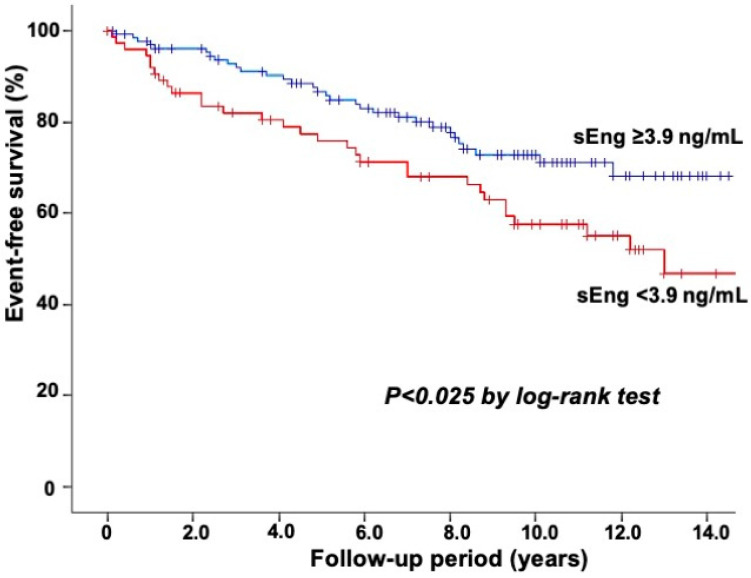
The event-free survival from cardiovascular events in 209 patients with CAD. Among the 209 patients with CAD, the Kaplan–Meier analysis also showed a lower event-free survival rate in patients with sEng levels <3.9 ng/mL than in those with ≥3.9 ng/mL (*p* < 0.025).

Furthermore, only among the 209 patients with CAD, 62 CAD patients with events had lower sEng levels (3.92 vs. 4.30 ng/mL) and more often had an sEng level <3.9 ng/mL (50% vs. 30%) than 147 CAD patients without events (*p* < 0.05). A Kaplan–Meier analysis also showed a lower event-free survival rate in patients with sEng levels <3.9 ng/mL than in those with ≥3.9 ng/mL (*p* < 0.025) (Figure 2). In the multivariate Cox regression analysis, the sEng level <3.9 ng/mL was an independent predictor of further cardiovascular events (HR: 2.07; 95%CI: 1.24–3.45, *p* < 0.01) in patients with CAD (Table 4). In the C-statistics analysis, the addition of sEng levels to the model including age, sex, hypertension, hyperlipidemia, stain use, smoking, diabetes mellitus, coronary revascularization at baseline, LVEF, and HDL cholesterol and hsCRP levels significantly improved the C-statistics from 0.56 (95%CI: 0.47–0.65) to 0.63 (95%CI: 0.54–0.71) (*p* < 0.05).

## 4. Discussion

The present study investigated the prognostic value of plasma sEng levels in 403 patients undergoing elective coronary angiography for suspected CAD. Plasma sEng levels were significantly lower in patients who developed cardiovascular events than in those who had no events. Low sEng levels were found to be associated with an increased risk of cardiovascular events, independent of CAD, LVEF, hsCRP levels, and atherosclerotic risk factors. Furthermore, low sEng levels were also an independent predictor of further cardiovascular events in 209 patients with CAD, and sEng levels improved the predictive accuracy for cardiovascular events, especially in patients with CAD. 

Regarding the association between blood sEng levels and atherosclerosis, hypercholesterolemia increased blood sEng levels and atherosclerotic lesions in ApoE/LDL-receptor knockout mice, and a decreased endoglin expression was simultaneously observed in the aortas of these mice [21]. Serum sEng levels were also reported to be high in patients with familial hypercholesterolemia [22]. Furthermore, serum sEng levels were reported to correlate positively with atherosclerotic risk factors and carotid intima-media thickness [23]. In contrast, endoglin expression was shown to be downregulated in monocytes of patients with familial hypercholesterolemia [8]. Regarding blood sEng levels in patients with CAD, Blann et al. [14] reported that there was no significant difference in serum sEng levels between 29 patients with CAD and 26 age- and sex-matched healthy controls. Interestingly, Li et al. [15] reported that serum sEng levels were lower, but the serum levels of endoglin/TGF-β complexes were higher in patients with three-vessel disease compared with the healthy controls. They suggested that low sEng levels in patients with CAD may be due to increased formation of endoglin/TGF-β complexes. As previously reported [13], the present study increased the number of study patients (from 244 to 403 patients) and also found that plasma sEng levels were significantly lower in 209 patients with CAD than in 194 without CAD. However, we were unable to measure the blood levels of endoglin/TGF-β complexes because of no commercially available kits to measure endoglin/TGF-β complex levels. Endoglin expression was shown to be upregulated in the vascular SMCs of human atherosclerotic plaques [10]. High expressions of both endoglin and TGF-β were also reported in the endothelial cells, SMCs, and macrophages of human atherosclerotic tissues [11]. It is still speculated that the early stage of atherosclerosis has increased sEng levels for endothelial damage, but the later stage of atherosclerosis has decreased sEng levels for increased endoglin/TGF-β1 complexes [5,15]. In spite of the increased endoglin expression in coronary atherosclerotic plaques [10,11], patients with CAD may have decreased blood sEng levels due to the increased formation of endoglin/TGF-β complexes.

Ikemoto et al. [24] investigated plasma sEng levels and cardiovascular events in 318 patients with CAD undergoing PCI, of whom only 27 patients had cardiovascular events during a mean follow-up period of 2.9 ± 1.7 years. Contrary to our results, they reported that patients with higher sEng levels more often had cardiovascular events. In spite of the use of the ELISA kit by the same manufacturer (R&D Systems) and the same ethnic population (Japanese), the cause of different results for us and Ikemoto et al. [24] remains unclear. However, consistent with our results, Cruz-Gonzalez et al. [25] reported that sEng levels in patients with acute MI were low and a greater sEng decrease over the first 48 h was associated with increased cardiovascular mortality. Moreover, serum sEng levels in patients with stroke tended to be lower than those in the healthy controls and decreased on day 6 after stroke [26]. Recently, Rossi et al. [27] reported that sEng inhibits platelet aggregation and thrombus formation in transgenic mice overexpressing sEng. They suggest that sEng is a decoy molecule that exerts an opposite effect to membrane-bound endoglin in thrombus formation and development [28]. Furthermore, Januszek et al. [29] investigated blood sEng levels in patients with peripheral artery disease undergoing a treadmill training program and reported that a high sEng level was a predictor of greater walking improvement. These findings thus suggest that lower sEng levels are associated with poor outcomes or cardiovascular events. In the present study, plasma sEng levels were significantly lower in 79 patients with cardiovascular events than in 324 without events, and lower sEng levels were associated with an increased risk of cardiovascular events in patients undergoing coronary angiography. Furthermore, even in 209 patients with CAD, a lower sEng level was also a significant predictor of cardiovascular events. Our results suggest that low, rather than high, sEng levels in the blood may be a biomarker for a high risk of cardiovascular events in patients undergoing coronary angiography and patients with CAD. However, endoglin is suggested to play an important role in balancing the proangiogenic and antiangiogenic responses of TGF-β [30]. Furthermore, high sEng levels with a high-fat diet was shown to induce the simultaneous activation of proinflammatory, oxidative, and vaso-protective mechanisms in the aorta of mice overexpressing sEng [31]. Therefore, further studies are warranted to elucidate the precise role and mechanism of blood sEng in the development of CAD and cardiovascular events and its prognostic value in patients with CAD and patients undergoing coronary angiography.

The present study had some limitations. First, the study population was relatively small (403 patients), and the number of patients with cardiovascular events was particularly low (79 patients). To validate the prognostic value of blood sEng levels in patients with CAD or those undergoing coronary angiography, further studies with a larger number of patients are needed. Furthermore, no patients with ACS were included in the present study. A further study on patients with ACS is needed to elucidate the prognostic value of sEng levels in ACS. Second, Kapur et al. [17] measured the serum sEng levels in 82 patients with a suspected LV dysfunction and reported that sEng levels were higher in patients with LV end-diastolic pressure (LVEDP) ≥ 16 mmHg, but not in those with LVEDP < 16 mmHg, than in the healthy controls. Although we excluded patients with any history of HF, we did not routinely measure the LVEDP in the coronary angiography and some of our study patients may have had high LVEDP levels. However, the sEng level was found to be a significant predictor of cardiovascular events independent of LVEF. Third, we performed a coronary angiography to assess the presence and severity of CAD. Angiography cannot observe coronary artery plaques and only shows the artery lumen characteristics. Furthermore, the degree of stenosis was not evaluated using quantitative coronary angiography (QCA) or coronary fractional flow reserve, but it was evaluated only by visual assessment with a single cardiologist, as in our previous study [13]. These may have confounded our results. Fourth, our study patients were Japanese who had a coronary angiography. Such patients were generally considered a highly selected population at a high risk for CAD. Our results may not be applied to the general population or other ethnic groups. Finally, we measured plasma sEng levels only at the baseline coronary angiography and did not evaluate any changes in the sEng levels during the follow-up period, which may have affected the outcomes. Furthermore, we did not evaluate any changes in drugs for the treatment of CAD, which may have affected the results of this study.

## 5. Conclusions

Plasma sEng levels were significantly lower in patients with cardiovascular events than in those without events, and lower sEng levels were found to be independently associated with an increased risk of cardiovascular events in patients with CAD as well as patients undergoing coronary angiography. Our results suggest that low sEng levels in the blood may be a biomarker for cardiovascular events.

## Figures and Tables

**Table 1:** Clinical characteristics and plasma sEng levels of patients with and without CAD.

	All			
	(n = 403)	CAD (−) (n = 194)	(−) vs. (+)	CAD (+) (n = 209)
Age (years)	67 ± 11	65 ± 12	*<0.001*	69 ± 10
Sex (male)	281 (70%)	120 (62%)	*<0.005*	161 (77%)
Hypertension	285 (71%)	119 (61%)	*<0.001*	166 (79%)
Hyperlipidemia	206 (51%)	78 (40%)	*<0.001*	128 (61%)
LDL cholesterol (mg/dL)	114 ± 31	111 ± 29	*NS*	116 ± 33
HDL cholesterol (mg/dL)	55 ± 15	58 ± 16	*<0.001*	51 ± 13
Statin use	144 (36%)	46 (24%)	*<0.001*	98 (47%)
Diabetes mellitus	99 (25%)	26 (13%)	*<0.001*	73 (35%)
Smoking	173 (43%)	68 (35%)	*<0.005*	105 (50%)
LVEF (%)	63 ± 10	63 ± 9	*<0.05*	61 ± 12
hsCRP level (mg/L)	0.63 [0.30, 1.53]	0.53 [0.27, 1.31]	*<0.01*	0.80 [0.37, 1.74]
**sEng level (ng/mL)**	4.33 [3.74, 5.06]	**4.41 [3.85, 5.18]**	***<0.025***	**4.26 [3.57, 4.97]**

Data are presented as the mean ± SD or number (%) of patients, with the exception of hsCRP and sEng levels, which are presented as the median value and interquartile range.

**Table 2:** Clinical characteristics and plasma sEng levels of patients with and without cardiovascular events.

	All			
	(n = 403)	Events (−) (n = 324)	(−) vs. (+)	Events (+) (n = 79)
Age (years)	67 ± 11	66 ± 11	*<0.005*	70 ± 11
Sex (male)	281 (70%)	222 (69%)	*NS*	59 (75%)
Hypertension	285 (71%)	223 (69%)	*NS*	62 (78%)
Hyperlipidemia	206 (51%)	161 (50%)	*NS*	45 (57%)
LDL cholesterol (mg/dL)	114 ± 31	112 ± 30	*NS*	119 ± 33
HDL cholesterol (mg/dL)	55 ± 15	55 ± 15	*<0.02*	51 ± 14
Statin use	144 (36%)	108 (33%)	*NS*	36 (46%)
Diabetes mellitus	99 (25%)	79 (24%)	*NS*	20 (25%)
Smoking	173 (43%)	133 (41%)	*NS*	40 (51%)
CAD	209 (52%)	147 (45%)	*<0.001*	62 (78%)
Number of stenotic vessels	1.0 ± 1.1	0.8 ± 1.0	*<0.001*	1.7 ± 1.1
LVEF (%)	63 ± 10	63 ± 9	*<0.05*	61 ± 12
hsCRP level (mg/L)	0.63 [0.30, 1.53]	0.60 [0.29, 1.49]	*<0.05*	0.82 [0.45, 1.86]
hsCRP > 1.0 mg/L	46 (36%)	113 (35%)	*NS*	33 (42%)
**sEng level (ng/mL)**	4.33 [3.74, 5.06]	**4.39 [3.83, 5.08]**	***<0.02***	**3.95 [3.49, 4.84]**
**sEng < 3.9 ng/mL**	129 (32%)	**92 (28%)**	***<0.005***	**37 (47%)**

Data are presented as the mean ± SD or number (%) of patients, with the exception of hsCRP and sEng levels, which are presented as the median value and interquartile range. The groups of Events (−) and Events (+) indicate patients who had no cardiovascular events and those who developed cardiovascular events, respectively.

**Table 3:** Factors associated with cardiovascular events. (A multivariate Cox proportional hazards analysis in the 403 study patients).

	Hazard Ratio	(95% CI)	*p* Value
Age (per 10 year increase)	1.38	(1.07–1.77)	*<0.02*
CAD	2.80	(1.61–4.88)	*<0.001*
LVEF (<50%)	2.80	(1.47–5.34)	*<0.005*
**sEng level (<3.9 ng/mL)**	**1.59**	**(1.01–2.49)**	***<0.05***

The dependent variable was cardiovascular events. This analysis included age, sex, hypertension, hyperlipidemia, stain use, smoking, diabetes mellitus, CAD, coronary revascularization (PCI or CABG) at baseline, LVEF (<50%), and HDL cholesterol (<40 mg/dL), hsCRP (>1.0 mg/L), and sEng (<3.9 ng/mL) levels.

**Table 4:** Factors associated with cardiovascular events. (A multivariate Cox proportional hazards analysis in the 209 patients with CAD).

	Hazard Ratio	(95% CI)	*p* Value
LVEF (<50%)	3.27	(1.62–6.62)	*<0.002*
**sEng level (<3.9 ng/mL)**	**2.07**	**(1.24–3.45)**	***<0.01***

The dependent variable was cardiovascular events. This analysis included age, sex, hypertension, hyperlipidemia, stain use, smoking, diabetes mellitus, coronary revascularization at baseline, LVEF (<50%), and HDL cholesterol (<40 mg/dL), hsCRP (>1.0 mg/L), and sEng (<3.9 ng/mL) levels.

## Data Availability

The data that support the findings of this study are available from the corresponding author on reasonable request.
